# Ultrasonographic Features of Muscular Weakness and Muscle Wasting in Critically Ill Patients

**DOI:** 10.3390/jcm13010026

**Published:** 2023-12-20

**Authors:** Michele Umbrello, Etrusca Brogi, Paolo Formenti, Francesco Corradi, Francesco Forfori

**Affiliations:** 1 Department Intensive Care and Anesthesia, ASST Ovest Milanese, Ospedale Nuovo di Legnano, 20025 Legnano, Italy; 2 Department Anaesthesia and Intensive Care, University of Pisa, 56126 Pisa, Italy; 3 Departement of Anesthesia and Intensive Care, ASST Nord Milano, Ospedale E Bassini, 20092 Cinisello Balsamo, Italy

**Keywords:** intensive care unit acquired weakness, muscle weakness, ultrasound evaluation, critical care, proteolysis

## Abstract

Muscle wasting begins as soon as in the first week of one’s ICU stay and patients with multi-organ failure lose more muscle mass and suffer worse functional impairment as a consequence. Muscle wasting and weakness are mainly characterized by a generalized, bilateral lower limb weakness. However, the impairment of the respiratory and/or oropharyngeal muscles can also be observed with important consequences for one’s ability to swallow and cough. Muscle wasting represents the result of the disequilibrium between breakdown and synthesis, with increased protein degradation relative to protein synthesis. It is worth noting that the resulting functional disability can last up to 5 years after discharge, and it has been estimated that up to 50% of patients are not able to return to work during the first year after ICU discharge. In recent years, ultrasound has played an increasing role in the evaluation of muscle. Indeed, ultrasound allows an objective evaluation of the cross-sectional area, the thickness of the muscle, and the echogenicity of the muscle. Furthermore, ultrasound can also estimate the thickening fraction of muscle. The objective of this review is to analyze the current understanding of the pathophysiology of acute skeletal muscle wasting and to describe the ultrasonographic features of normal muscle and muscle weakness.

## 1. Introduction

Patients discharged from the ICU very commonly present severe muscle dysfunction leading to the inability to accomplish daily activity and suffer from a reduced quality of life as a consequence [1,2]. Indeed, critically ill patients may present several complex symptoms and physical findings that are responsible for physical impairments, cognition difficulties, and mental health alterations. Post-ICU syndromes have a huge impact during the recovery phases of patients and caregivers. Disabilities can last up to 5 years and almost half of patients are not able to return to work during the first year after ICU discharge. This limitation in working activity and difficulties in performing activities of everyday life lead to a reduced quality of life with important economic challenges for both patients and their families [3].

During ICU stay, muscle mass rapidly deteriorates in the early phases of critical illness, with sequelae that may persist also during the recovery phases (e.g., muscle deficit, falls) [4,5,6,7]. Muscle wasting can be triggered by several factors and its severity is directly correlated with the gravity of the underlying critical illness [1]. In 2014, the American Thoracic Society defined intensive care acquired weakness (ICUAW) as “a syndrome of generalized limb weakness that develops while the patient is critically ill and for which there is no alternative explanation other than the critical illness itself” [8]. Remarkably, the respiratory and oropharyngeal muscles can also be affected: aspiration pneumonia and dysphagia represent well-known and possible complications of such muscle wasting syndrome [9,10,11,12].

ICUAW is characterized by heterogenous clinical presentation with different impairments in muscle contractility and strength [13,14]. Proteostasis and, consequently, the pathways involved in the homeostasis of protein synthesis and degradation play a central role in the pathophysiology of muscle wasting syndrome. However, the detailed mechanism involved remains only partially understood [15]. In recent years, ultrasound has gained more and more importance in the evaluation of skeletal muscle, both quantitatively and qualitatively. Ultrasound allows the evaluation of the cross-sectional area and thickness of the muscles as indices of muscle mass. Moreover, ultrasound provides clinicians with important information on the echogenicity and the presence of muscle atrophy [16]. Remarkably, ultrasound can also be used for the assessment of the diaphragm [17,18,19].

In this review, we aimed to summarize the current understanding of the pathophysiology of acute skeletal muscle wasting, describe the ultrasonographic (US) features of normal muscle, and summarize the US assess muscle weakness.

## 2. Pathophysiology of Muscle Weakness and Muscle Wasting during Critical Illness

In critically ill patients, the loss of muscle mass and strength predominantly results from the imbalance between protein breakdown and synthesis, with increased protein degradation relative to protein synthesis. The resulting net catabolic state is responsible for ICUAW. An overview of the mechanisms involved in the pathophysiology of such acute skeletal muscle wasting is shown in Table 1.

Malnutrition and immobility have a huge impact on the short- and long-term changes of body composition, leading to functional debility and an increased risk of fractures. Furthermore, various concomitant factors can lead to bioenergetic dysfunction and altered protein synthesis. Above all, sepsis, oxidative stress and burns represent critical risk factors for muscle wasting [1,5,20]. A systemic inflammatory response is highly prevalent in critically ill patients, especially during the acute phase, and it can persist during the recovery phase with potential risks for chronic critical illness and with huge resulting negative impacts on post-ICU recovery. From a pathophysiological point of view, the systemic inflammatory response is characterized by the mismatch between oxygen supply and demand leading to mitochondrial bioenergetic disequilibrium [16]. The consequent decrease in ATP production leads to reduced protein synthesis. Moreover, whilst in an inflammatory state, increased capillary permeability microcirculatory impairments, endothelial dysfunction lead to reduced nutrient delivery, the effusion of toxic molecules, and cell damage with the resulting increased protein degradation [21]. It is worth noting that several studies have shown a direct correlation between disease severity, inflammatory mediators’ levels (e.g., tumor necrosis factors) and the degree of muscle atrophy, axonal degeneration, and loss of myelinated fibers [5,20,22,23,24].

Major signaling pathways that control muscle growth are implied in the muscle wasting syndrome. Both the insulin-like growth factor 1 phosphoinositide-3-kinase-Akt/Protein kinase B mechanistic target of rapamycin (IGF1-PI3K-Akt/PKB-mTOR) pathway and the myostatin-Smad 3 pathway represent two key signal networking which regulate muscle growth. The IGF1-PI3K-Akt/PKB-mTOR pathway regulates several central metabolic mechanisms such as protein synthesis, protein degradation, glucose uptake, energy production, and cellular proliferation. Under the action of growth hormones, IGF1 is predominantly synthesized in the liver and is responsible for activating the mitogen-activated protein kinase/extracellular signal-regulated kinase (RAS-MAPK-ERK) and the PI3K–AKT-mTOR pathways. The kinase mTOR interacts with several molecules (such as hormones and cytokines) and forms two complexes. In detail, the mTORC1 complex mainly regulates cell growth, controlling protein synthesis and mitochondrial biogenesis, whereas the mTORC2 complex is implied in glucose and lipid homeostasis. mTOR controls both the anabolic and catabolic state with a central role in energy sensing and the modulation of muscle growth. Consequently, the dysregulation of mTOR signaling may lead to metabolic disorders, cancer, and neurodegeneration. The nutrient level, growth factors, as well as the energy and oxygen availability all represent regulators of mTORC1 expression, with resulting implications for translation and spliced transcription, ribosome biogenesis, nucleotide synthesis, autophagy, and lysosome biogenesis. An important consequence in the alteration of these signaling pathways is represented by the reduced mRNA expression for myosin-heavy chains at the translational level [25].

Protein homeostasis, also known as proteostasis, is fundamental in physiological conditions to maintain normal muscle health and prevent several illnesses. As proteostasis involves a balance between protein synthesis and degradation, cellular proteolysis systems are vital for preserving cellular functioning. The ubiquitin (Ub)/proteasome system (UPS), the autophagy/lysosomal system, and the caspase-mediated protein cleavage represent the systems involved in the proteolytic pathways which are, consequently, involved during muscle wasting [25]. The ubiquitin (Ub)/proteasome system is responsible for protein degradation control, and it regulates cellular processes such as DNA repair, stress response, and cell proliferation. Proteasome (i.e., 26S proteasome) is a proteolytic protease that degrades proteins through an ATP-dependent process. To be recognized by the proteasome, proteins have to be tagged by the E1 ubiquitin-activating enzyme, E2 ubiquitin-conjugating enzymes, and E3 ubiquitin–protein ligases. Several pro-inflammatory stimuli can increase UPP gene transcription, protein levels of ubiquitin ligases as well as proteosome activity, thus accelerating muscle catabolism [26]. FoxO1, FoxO3, atrogin-1, MuRF1 and 2, FBOX31, SMART, and TRIM 32 represent important Ub ligases implied in proteolytic process during muscle wasting. Atrogin-1 and MuRF1 are E3 ubiquitin ligases with a particular interest. Importantly, glucocorticoids can activate these ligases, leading to the protein degradation and release of amino acids that represent important sources for gluconeogenesis and consequently induce atrophy [27]. The second main proteolytic system controller in response to cell stress is represented by autophagy [28]. Autophagy is upregulated by fasting, ROS, inflammation, growth factors and infection. Both the upregulation and dysregulation of autophagy are responsible for increased muscle degradation [29]. Important molecules that regulate autophagy are: FoXO, mTORC1, LC3, and Atg7. Heat shock proteins are upregulated on the first day of ICU admission.

Eventually, glucose and calcium metabolism represent further central mechanisms implied in muscle wasting syndrome. Hyperglycemia is tightly linked to exaggerated inflammatory responses, immune system imbalance, and mitochondrial damage, leading to peripheral neuropathies and respiratory muscle weakness [30]. High glucose levels can activate the ubiquitin–proteasomal degradation pathway, and can increase the production of reactive oxygen species which in turn increase the proteolytic pathways [31]. It is worth noting that glucose can play a direct cellular toxic effect on the cells due to a hyperosmolar hyperglycemic state. This mechanism also contributes to protein degradation and cellular necrosis. Additionally, the calcium levels are implied in the upregulation of calpain and consequently trigger the proteolysis pathway [32]. Indeed, calpains are cysteine proteases with proteolysis effects that result in actin and myosin myofibril degradation. Furthermore, increased levels of calcium in the cytosol can be toxic, promoting ROS production and apoptosis [33,34]. In addition, it is worth highlighting that the Na^+^/K^+^ pump exchangers play a central role in the process of membrane action potential and in the regulation of intracellular calcium levels. Altered channelopathy is frequent during sepsis with consequent membrane depolarization and denervation effects with altered muscle contraction function [33].

## 3. Ultrasound Features of the Normal Muscle

Muscles consist of two components: the muscle fibers and the stromal connective tissue. Muscle comprises muscle fibers organized in fascicles surrounded by a connective tissue, called perimysium [35]. Within the fascicles, multiple groups of fibers are covered by endomysium. Epimysium represents the outermost sheath of connective tissue covering the muscle. Inside the three bundles of muscle fibers, blood vessels, nerve fibers, subcutaneous fat and connective tissue are present [36]. Skeletal muscles differ in size, shape, and fibers disposition. Fiber can be parallel to the longitudinal axis of the muscle (running for the entire length of the long axis of the muscle), or they can present an oblique disposition or converge to a specific point [37]. Fusiform muscle bundles have a parallel orientation in the median portion of muscle so that the fibers converge towards the tendon at the muscle ends, whereas pennate muscles are characterized by an oblique fascicular arrangement of the fibers relative to the line of pull [38]. In bipennate muscles, the fascicles converge into a single central tendon, while multipennate muscles show more than one tendon running through the muscle substance [39]. Furthermore, the fibers can converge to a fibrous apex through a wide attachment (fan-shaped or triangular muscle) [40].

Ultrasound is a dynamic technique that is therefore capable of visualizing normal and pathological muscle features. US is generally performed using high-frequency probes, allowing a better lateral resolution, axial resolution, and a reduction in distortion in comparison with curved array probes [41].

Normal ultrasonographic features of muscle show an organized structure allowing the transmission of sound waves [42]. Ultrasonographic evaluation can discriminate structures such as subcutaneous fat, bone, nerves, and blood vessels [43]. Moreover, dynamic ultrasound imaging, using high frame rates, can analyze muscle contractions, tremors, and fasciculations [44]. The echotexture of normal skeletal muscles is characterized by a hypoechoic texture reflecting the demarcated linear hyperechoic muscle bundles and filaments (i.e., “starry night” appearance); this speckled appearance represents the reflections of perimysial connective tissue in the short-axis plane [45,46]. In the longitudinal plane, it is possible to visualize the general architecture of the muscle with the fibers’ disposition. Consequently, in this view, it is possible to appreciate the organization of the different fibers (i.e., parallel in the biceps brachii, pinnate in tibialis anterior, and triangular, as in the latissimus dorsi muscle) [47]. Perimysium reflections result in a linear, pennate, or triangular structure image. In both the US views, along or perpendicular to the long axis of the muscle, epimysium surrounding the muscle is clearly identifiable as a highly reflective structure as well as the bone with a hyperechogenic rim and an anechoic bone shadow underneath [48]. The proportion in connective tissue and muscle fascicles can be analyzed by the evaluation of the ratio between the hypoechoic and hyperechoic components of the muscle [49]. Nerves, intramuscular tendons, and aponeuroses are relatively hyperechoic bands whereas subcutaneous fat has a low echo intensity. In detail, tendons look like fibrillar structures with multiple stripes‘ organization.

The evaluation of muscle size, area, and changes in echo intensity pattern and echotexture provides important information on the health of the muscle [50]. Furthermore, ultrasound supports the evaluation of post-traumatic muscle complications and the presence of muscle edema. Reflection, absorption, and attenuation depend on the composition of the muscle that can vary hugely during neuromuscular disease and trauma [51,52]. Indeed, under physiological conditions, the proportion of fibrous tissue is low; consequently, this results in a hypo-anaechogenic image. In the evaluation of muscle thickness and of the cross-sectional areas, the operator has to take into account the variety encountered across different ages. Actually, age influences muscle thickness; size increases rapidly during childhood, with a peak between 25–50 years of age; and then declines progressively [53]. The MRI studies have shown a good correlation between the determination of muscle thickness using US and magnetic resonance evaluation (i.e., Pearson coefficient r = 0.99, *p* < 0.01) [54,55,56,57]. Further important US evaluation is represented by echogenicity [45].

The proportion of fibrous tissue as well as fiber arrangement can influence the echogenicity [58]. When US waves meet a tissue characterized by varying acoustic impedance, part of the acoustic waves is reflected; the amplitude of the reflected acoustic signal determines the brightness of the displayed image. Indeed, fibrous tissue presents a different acoustic impedance in comparison to muscle fibers, with high absorption and attenuation, resulting in a brighter image [42]. Discontinuity in muscle density results in a reflection and scattering phenomenon. Consequently, echogenicity increases with age due to the increased proportion of fat and fibrous tissue within the muscle [59]. Additionally, muscular dystrophy is characterized by an alteration in muscle size and an increased proportion of fat and connective tissue; in these cases, a “ground glass” appearance is observed due to increased echogenicity [60]. Likewise, US can analyze the structured organization of fibers; this arrangement can be lost in neuromuscular diseases with a “patchy” or “steaky” appearance [60]. Echogenicity can be evaluated using qualitative methods. Heckmatt scale is a four-point visual grading scale based on gray-scale appearance [47]. This visual evaluation scale has shown high sensitivity and high inter-observer agreement [61]. Interestingly, echogenicity can also be evaluated using quantitative methods. Acquisition tools allow the acquisition of the mean gray scale level of a region of interest (ROI) and the comparison with a mean reference value of echogenicity for the muscle [45,62,63]. In addition, spatial frequency analysis (SFA) can evaluate the B-mode speckle pattern of muscle in the spatial frequency domain providing important information on muscle architecture [64].

**Figure 1:**
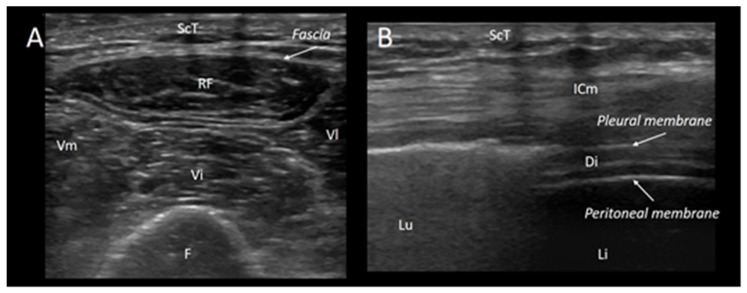
Normal features of diaphragm and quadriceps muscle. The figure shows the features that can be seen with skeletal muscle ultrasound. Left Panel (**A**): a transverse scan of the thigh allows for the visualization of the quadriceps muscle, which is composed of the rectus femoris (RF), the vastus lateralis (Vl), the vastus medialis (Vm), and the vastus intermedius (Vi); the hyperechoic, curvilinear image on the lower part of the figure, with the anechoic shadow is the femur (F), while on the upper part of the image, the subcutaneous tissue is shown (ScT). Right panel (**B**): a longitudinal scan of the chest wall at the zone of apposition allows for the visualization of the diaphragm at the zone of apposition (i.e., the area of attachment between the diaphragm directly behind the inner aspect of the lower chest wall and rib cage). Lu: lung; ScT: subcutaneous tissue; ICm: intercostal muscles; Li: liver; and Di: diaphragm.

Finally, the US can allow the visualization of muscle movement in real time [65]. This aspect is remarkable, taking into consideration that US can analyze the diaphragm movement through the respiration cycle [66]. During inspiration, diaphragm thickness increases up to 20% from FRC to TLC [67]. Even more, the bilateral evaluation of the diaphragm can allow the detection of muscle asymmetry in the case of unilateral phrenic palsy [68]. Diaphragmatic ultrasound evaluation has shown a correlation with MIP and with the phrenic nerve conduction study. Diaphragmatic US evaluation has also shown a high sensitivity and specificity for the detection of neuromuscular diaphragmatic weakness characterized by a reduction in thickness and muscle excursion during inspiration [69]. Remarkably, the US can also allow the identification and the extent of fasciculations [65,70]. Tremors and fibrillations can also be seen. Fasciculations are a marker of denervation and the detection of fasciculation represents a vital finding for amyotrophic lateral sclerosis’ diagnosis [71,72]. Likewise, fibrillation indicates a loss of interaction between the muscle and innervation axon, and it can be seen in several muscular disorders. Consequently, the ultrasound evaluation of muscle contraction can represent a huge aid for clinicians with the diagnosis of neuromuscular disease [65]. Figure 1 shows the features that can be seen with skeletal muscle ultrasound.

## 4. Ultrasonographic Evaluation of Muscle Wasting in the Critically Ill

The ultrasonographic evaluation of muscle wasting is a valuable tool in the assessment and management of critically ill patients [73]. Muscle wasting and atrophy are common problems in the critically ill due to various factors, including prolonged bed rest, immobilization, systemic inflammation, and the catabolic response to stress [74,75,76]. When assessing muscle wasting, clinicians select specific muscles based on clinical relevance and accessibility. The most commonly evaluated muscles are in the limbs and include the quadriceps, rectus femoris, and the anterior compartment muscles of the thigh [61,77,78,79]. Ultrasonography typically involves using a high-frequency ultrasound probe oriented to obtain longitudinal or transverse images of the muscle of interest. Muscle thickness is often measured at specific anatomical landmarks, such as the midpoint or lower third of the thigh for quadriceps assessment [80] or the 10th intercostal space on the anterior axillary line for diaphragm ultrasound [81]. The cross-sectional area of the rectus femoris muscle is determined by outlining the muscle borders on the ultrasound image, and the software calculates the area based on the traced boundaries [82,83]. It is well known that the limb muscle size, structure, and function deteriorate during the course of critical illness, by approximately 3% per day in the first week of ICU stay [84] and ultrasound findings of reduced rectus femoris CSA were found to be associated with poor clinical outcomes [85]. Similarly, a quality assessment can be obtained by the analysis of muscle echogenicity, that is, the ability to reflect or transmit ultrasound waves within the context of surrounding tissues. Echogenicity is currently measured offline on saved, exported images, by performing the grey-scale analysis of image pixels using standard software for image editing. In healthy muscles, echogenicity is relatively uniform and appears hypoechoic (i.e., darker) on ultrasound images. Muscle degeneration and fatty infiltration result in increased echogenicity, leading to a hyperechoic (brighter) appearance. This process has been shown to correlate with ultrastructural findings, as it reflects the muscle composition: increased echogenicity represents a more homogenous muscle [22]. The quantification of muscle echogenicity requires exporting the muscle ultrasound scan as a digital image file for subsequent, offline computer analysis, and the absolute value of density of the image critically depends on the settings which the image was acquired with. A recent investigation in healthy volunteers has analyzed vastus lateralis ultrasound images at increasing depths from 3 to 7 cm and gain settings of 50 and 60 dB. The authors found that echo intensity values were similar between 4 and 6 cm regardless of the gain, suggesting that, regardless of the image gain, a stable depth and gain setting should be used, even if small deviations may be acceptable [86]. Changes in quadriceps muscle echogenicity have been associated with negative outcomes [22].

On the other side, diaphragm ultrasound has also been used, besides the evaluation of the patient contribution to the inspiratory work of breathing, to study muscle wasting [87]. To do so, the end-expiratory thickness is generally used, as its value is not influenced by the inspiratory thickening secondary to muscle contraction. In healthy subjects, an end-expiratory thickness of 1.7–2.2 mm is expected [88]. In invasively ventilated patients, the diaphragm thickness was shown to be a reliable measure of weakness, in terms of delayed ventilator weaning [89].

The functional assessment of rectus femoris can involve dynamic ultrasound imaging during muscle contraction [90]. For example, the quadriceps can be assessed for its ability to contract and generate force during a leg lift. The clinician observes muscle movement and changes in thickness as the muscle contracts and relaxes. Similarly, muscle architecture can be described by the pennation angle, i.e., the angle of insertion of muscle fibers into their aponeurosis, which provides information about muscle strength. For instance, the larger the pennation angle, the more contractile material is present, and thus the higher the capacity is to produce force [58]. The rectus femoris pennation angle is measured with the same method and in the same position of muscle area and thickness; a longitudinal view is obtained by rotating the probe parallel to either the lateral or medial head of the muscle. A few studies have investigated the pennation angle in critically ill subjects; showing how, upon ICU admission, an angle < 4.4° was found to be associated with a worse outcome [91]. Serial ultrasound assessments can be performed at regular intervals (e.g., weekly or monthly) to track changes in muscle size and quality [92]. Trend analysis involves plotting these measurements over time to visualize the progression of muscle wasting [93]. Then, ultrasound findings can be integrated with nutritional assessments, including the measures of protein intake and serum albumin levels [94]. More specifically, the ESPEN suggest how ultrasound can evaluate a low body mass index, unintentional loss of body weight, low skeletal muscle mass index, decreased food intake/assimilation, and disease burden/inflammation, all of which are essential items for nutritional assessment [95]. In COVID-19, critically ill patients, early changes in muscle size and quality were shown to be related to ICU survival, and to be influenced by nutritional and fluid management strategies [96]. If muscle wasting is determined to be related to malnutrition, a tailored nutritional plan, possibly including protein supplementation, may be initiated. A recent pilot study [97] showed how muscle thickness assessed by ultrasonography independently predicts one’s nutritional status, similarly to previous studies in which the authors showed a moderate positive correlation between the rectus femoris and geriatric nutritional risk index [98]. The early detection of muscle wasting through ultrasound allows for timely intervention [78]. Physical therapists can design specific exercise programs to target the affected muscles. Rehabilitation plans can be adjusted based on the serial ultrasound assessments to optimize muscle recovery. Thus, the detailed documentation of ultrasound findings, including images and measurements, is crucial for clinical records and multidisciplinary collaboration.

Nevertheless, critical care muscle ultrasound research is hampered by several factors including the absence of a formal training program or standardized protocol used to educate clinicians, healthcare providers, or students; a lack of standardization in terms of specific muscles and muscle groups to be analyzed; and the absence of a standardized number of images needed for the adequate analysis of biomechanical measures and metabolic integrity.

**Figure 2:**
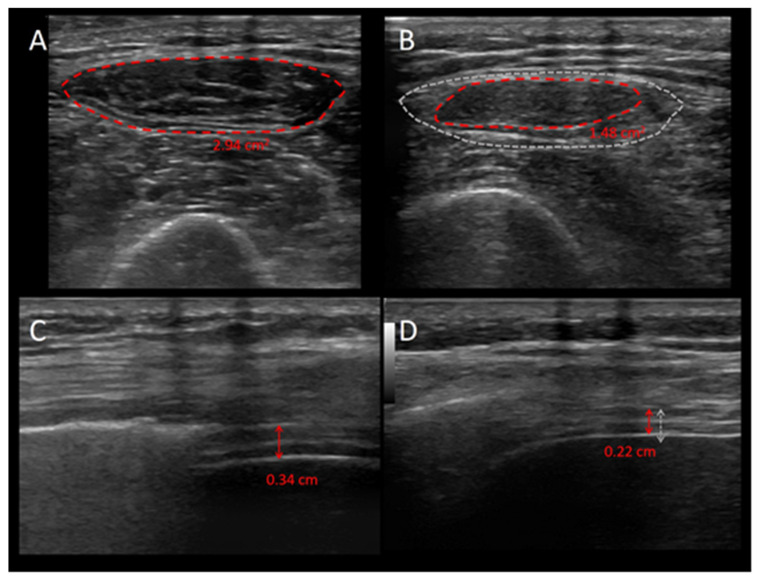
Ultrasonographic images of the quadriceps muscle and the diaphragm during the first week of ICU stay of a critically ill patient. The figure shows the images of the quadriceps muscle (upper panels (**A**,**B**)) and the diaphragm (lower panels (**C**,**D**)) of a critically ill patient during the first (left panels) and seventh day of ICU stay (right panels) and highlights the loss in muscle mass. Upper panels: The red, shaded line represents the cross-sectional area of the rectus femoris muscle; the grey, shaded area in panel (**B**) is the cross-sectional area of the first ICU day, and highlights the reduction in mass of the muscle. Lower panels: the red arrows show the diaphragm end-expiratory thickness; the grey, shaded line in panel (**D**) is the end-expiratory diaphragm thickness of the first ICU day.

In summary, the ultrasonographic evaluation of muscle wasting in critically ill patients is a precise and versatile approach that combines various parameters to assess muscle size, quality, and function. This detailed assessment aids in diagnosing and managing muscle wasting, ultimately contributing to improved patient outcomes and recovery in the critical care setting. Figure 2 shows the ultrasonographic images of the diaphragm and quadriceps muscle for a critically ill patient during the first week of ICU stay, and highlights the reduction in the mass of both muscles.

## 5. Ultrasonographic Assessment of Muscle Weakness

The ultrasonographic assessment of muscle weakness is a valuable diagnostic tool used to evaluate muscle function, size, and structure. Ultrasound is increasingly being used to assess changes in muscle size and quality over time. The advantages are the high spatial resolution, low procedural risks, absence of ionizing radiation, and ease of use, even early in the course of disease. Commonly assessed muscles include the quadriceps, biceps brachii, gastrocnemius, and others relevant to the patient’s condition [99], even if the most common site for muscle ultrasound is the rectus femoris [100]. In fact, this muscle is easy to identify and analyze with a single image, and is considered a functionally important muscle for the performance of daily living, while at the same time, being an antigravity muscle, it is subject to significant wasting during bedrest and illness, more than the muscles of the upper limbs [101]. The ultrasound probe is positioned over the target muscle, ensuring good skin contact and adequate gel application to optimize the image quality. The muscle thickness and cross-sectional area are primary parameters measured during the ultrasonographic assessment. These measurements are taken in both relaxed and contracted states to assess the muscle function. Measurements are usually obtained at standardized anatomical landmarks, such as the midpoint or two-thirds of the distance between the anterior superior iliac spine and the superior border of the patella. The rectus femoris ultrasound is generally performed using a high-frequency, linear transducer array probe (8–12 MHz), using the B-mode setting. Briefly, patients are studied in the semi-recumbent position with extended knees. The probe is placed on the anterior part of the thigh, on an imaginary line connecting the anterior superior iliac spine and the midpoint of the proximal border of the patella. A mark can be drawn on the skin to increase the reproducibility of subsequent measurements. The transducer is oriented in transverse to the longitudinal axis of the thigh at a 90° angle; the probe is coated with water-soluble transmission gel to increase the acoustic contact, and care is taken to reduce the pressure on the tissues and the consequent distortion of the image as much as possible. Typical values of the quadriceps thickness and rectus femoris CSA in healthy volunteers have been reported to be 2.6 cm and between 4.53 and 8.68 cm^2^ [102], respectively. On the other side, in critically ill patients, average values at ICU admission have ranged between 0.98 and 2.23 cm for quadriceps thickness and from 2.26 to 4.42 cm^2^ for rectus femoris CSA [22,103]. Such widely scattered values depend on the lack of a universally standardized technique for performing muscle ultrasound.

**Figure 3:**
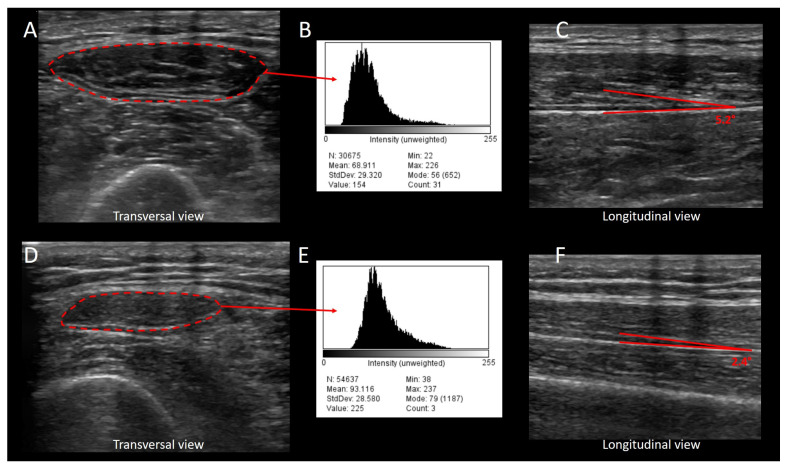
Change in rectus femoris echogenicity and pennation angle for a critically ill patient during the first week of ICU stay. The figure shows images of the quadriceps muscle of a critically ill patient during the first (upper panels) and seventh day of ICU stay (lower panels), and highlights the loss in muscle architecture. (**A**): the rectus femoris muscle is insonated with a transversal scan of the thigh; the red, shaded, line represents the cross-sectional area of the rectus femoris muscle; (**B**): the pixel enclosed in the red, shaded area of the rectus femoris cross-sectional area in panel (**A**) was analyzed for echo-genicity; (**C**): the rectus femoris muscle is insonated with a longitudinal scan at the same point of image (**A**); the pennation angle is shown by the red lines. Images (**D**–**F**) represent the same muscle of the same patient after 7 days of ICU stay. In the late image (panel (**E**)), the overall histogram is shifted to the right compared to the admission data (Panel (**B**)); moreover, the mean echo-intensity value increases from 68.9 to 93.1, indicating a brighter muscle, which is generally what happens with in-flammation or edema.

To identify muscle weakness, the thickness or cross-sectional area of the affected muscle is compared to the contralateral, unaffected side, or to established normative values. This comparison helps quantify the degree of muscle atrophy or weakness. Ultrasonography allows for the assessment of muscle quality by evaluating echogenicity, as mentioned above. Basically, once selected and saved, the appropriate ultrasound images should be exported in JPEG format. Then, the echogenicity can be quantified using a greyscale histogram analysis using the square method [104]. This allows one to define the region of interest for analysis using the histogram function, selecting a free-form area devoid of artefacts. A recent investigation in 37 critically ill subjects, 24 of whom developed ICU-acquired weakness, found that the changes in the thickness and CSA of various skeletal muscles between admission and the 10th day of ICU stay was associated with the development of weakness with areas under the ROC curves between 0.734 and 0.888 [92]. A subsequent study in 31 critically ill patients confirmed how a decrease in rectus femoris CSA > 10% during the first 72 h after ICU admission increased the risk for limb muscle weakness and handgrip weakness [105]. A recent systematic review and meta-analysis that included chronic as well as acutely ill subjects (including critically ill patients) found that muscle thickness, CSA, and echo-intensity have been shown to be correlated with functional outcomes such as muscle strength, physical function, exercise tolerance, and quality of life [106]. The pennation angle represented the angle of insertion of muscle fascicles into the deep aponeurosis, which is typical of bipennate muscles such as quadriceps. The pennation angle of the rectus femoris can be measured on the longitudinal view by orienting the probe parallel to either the lateral or medial head of the muscle. This method measures the fascicle closest to the widest point in the muscle to minimize variation in the pennation angle in just one muscle. As tension increases in the muscle fibers, the pennation angle also increases. Thus, a greater pennation angle results in a smaller force being transmitted to the tendon. Recent observations showed how the reduction in the pennation angle may offer high diagnostic accuracy for ICUAW, enabling an earlier diagnosis before patients become able to perform volitional tests [107]. Figure 3 shows the change in rectus femoris echogenicity and pennation angle for a critically ill patient during the first week of ICU stay, and highlights the muscle architectural deterioration.

In summary, the features of skeletal muscle, which include muscle quantity measures like mass and cross-sectional area and muscle quality measures such as architecture and evidence of myonecrosis, may provide a more feasible and objective approach to assessing muscle health in ICU patients. Objective quantifications of muscle (including muscle mass, thickness, and cross-sectional area) that are sufficiently sensitive to detect small changes over acute timeframes may eventually facilitate the evaluation of interventions to counter muscle atrophy and weakness.

## 6. Conclusions

In conclusion, muscle wasting is an early and significant manifestation of critical illness, and is associated with a high prevalence of weakness which may last long after the resolution of critical illness and ICU discharge. Ultrasound is the most useful bedside diagnostic tool available to evaluate the loss in muscle mass over the course of critical illness, while at the same time, it has been shown to be accurate and reproducible. While several features of muscle mass and architecture can be evaluated with ultrasound, we currently lack validated and widely used intervention protocols based on such measurements to monitor for the occurrence of muscle wasting or to guide the therapy. Hence, the dissemination of these measures is still limited in the critical care setting. Further studies are therefore of essential importance to better establish the role and position of these instruments in the context of the diagnostic–therapeutic pathway of critically ill patients.

## Figures and Tables

**Table 1:** Pathophysiology of muscle wasting, ICU-AW.

**Muscle Protein Synthesis**		
**Pathway**	**Mechanism**	**Effects**
IGF1-PI3K-Akt/PKB-mTOR pathway	- Regulated by mechanical load, nutrition, and growth factors; - IGF1 is predominantly synthesized in the liver; - Activates RAS-MAPK-ERK and the PI3K–AKT-mTOR pathways.	- Reduced expression of the mRNA; - Reduced myosin heavy-chain synthesis; - Control protein synthesis; - Control mitochondria biogenesis; - Metabolic disorder, cancer and neurodegeneration.
**Muscle protein breakdown**		
**Proteolytic system**	**Mechanism**	**Effects**
Ubiquitin (Ub)/proteasome system (UPS)	- Activation by Inflammatory factors and glucocorticoids; - Proteins are tagged by E1 ubiquitin-activating enzyme, E2 ubiquitin-conjugating enzymes, and E3 ubiquitin–protein ligases; - Complex recognized and degraded by 26S proteasome.	- Protein degradation control; - Regulates cellular processes: DNA repair and cell proliferation; - Degrading defective proteins.
Autophagy	- Chaperon-mediated autophagy; - Lysosomal pathway of proteolysis; - Microautopaghy; - Macroautophaghy.	- Clearance of damaged proteins/organelles; - Degradation of cytosolic protein; - Vacuolization of myofibers and nuclei; - Accumulation of p62 and ubiquitinated proteins; - Muscle atrophy.
Calpain and Caspase-3	- Increase during fasting; - Caspase -3 degrade endogenous calpain inhibitor; - Calpain facilitate Caspase-3 activation; - Calpain degrades structural proteins; - Caspase-3 degrade actomyosin.	- Proteolysis; - Mediate degradation of the myofibrillar apparatus; - Digestion of individual myofibrillar proteins; - Disassembly of the myofibril; - Initiation and regulation of cell death.
**Glucose metabolism**		
**Mechanism**		**Effects**
- Glycogenolysis and gluconeogenesis; - Insulin resistance; - Reduced glucose transporters (GLUT4) expression in muscle cells; - Activation of caspase 3, and the ubiquitin–proteasomal degradation pathway; - Direct glucose cellular toxicity.		- Release of glucose; - Activation protein degradation leading to muscle atrophy; - Increased production of reactive oxygen species; - Accumulation of sorbitol and fructose; - Intracellular hyperosmotic state; - Cell swelling and necrosis.
**Calcium level and channelopathy**		
**Mechanism**		**Effects**
- Upregulate calpain expression; - Altered ATP production; - Altered receptors and ion channel function → abnormal calcium release.		- Activation of proteolysis pathway; - Actin and myosin myofibril degradation; - Disruption of myofilament structure; - Reduced contractility and force generation.

## Data Availability

No new data were created or analyzed in this study. Data sharing is not applicable to this article.

## Abbreviation

ICU	Intensive care unit
ICU-AW	Intensive care-acquired weakness
US	Ultrasound
ATP	Adenosin Triphosphate
IGF1-PI3K-Akt/PKB-mTOR	Insulin-like growth factor 1 phosphoinositide-3-kinase -Akt/protein kinase B mammalian target of rapamycin
RAS-MAPK-ERK	Mitogen-activated protein kinase/extracellular signal-regulated kinase
mTORC1	Mammalian target of rapamycin complex 1
mTORC2	Mammalian target of rapamycin complex 2
mRNA	Messenger ribonucleic acid
UPS	Ubiquitin (Ub)/proteasome system
DNA	Deoxyribonucleic acid
ROS	Reactive oxygen species
MRI	Magnetic resonance imaging
FRC	Functional residual capacity
TLC	Total lung capacity
MPI	Maximum inspiratory pressure
ROI	Region of interest
SFA	Spatial frequency analysis
ESPEN	The European Society for Clinical Nutrition and Metabolism
COVID	Coronavirus disease
